# The Role of Neighborhood Characteristics in Late Stage Melanoma Diagnosis among Hispanic Men in California, Texas, and Florida, 1996–2012

**DOI:** 10.1155/2017/8418904

**Published:** 2017-06-18

**Authors:** Valerie M. Harvey, Clinton W. Enos, Jarvis T. Chen, Hadiza Galadima, Karl Eschbach

**Affiliations:** ^1^Department of Dermatology, Eastern Virginia Medical School, Norfolk, VA, USA; ^2^Hampton University Skin of Color Research Institute, Hampton, VA, USA; ^3^Department of Social and Behavioral Sciences, Harvard T.H. Chan School of Public Health, Boston, MA, USA; ^4^Center for Health Analytics Discovery and Graduate Program in Public Health, Eastern Virginia Medical School, Norfolk, VA, USA; ^5^Sealy Center on Aging and Center to Eliminate Health Disparities, University of Texas Medical Branch, Galveston, TX, USA

## Abstract

**Background:**

Hispanics diagnosed with cutaneous melanoma are more likely to present at advanced stages but the reasons for this are unknown. We identify* census tracts *at high risk for late stage melanoma diagnosis (LSMD) and examine the contextual predictors of LSMD in California, Texas, and Florida.

**Methods:**

We conducted a cross-sectional study using geocoded state cancer registry data. Using hierarchical multilevel logistic regression models we estimated ORs and 95% confidence intervals for the impact of socioeconomic, Hispanic ethnic concentration, index of dissimilarity, and health resource availability measures on LSMD.

**Results:**

We identified 12,493 cases. In California, late stage cases were significantly more likely to reside within census tracts composed mostly of Hispanics and immigrants. In Texas, LSMD was associated with residence in areas of socioeconomic deprivation and a higher proportion of immigrants. In Florida, living in areas of low education attainment, high levels of poverty, and a high percentage of Hispanic residents was significantly associated with LSMD. Residential segregation did not independently affect LSMD.

**Conclusion:**

The influence of contextual predictors on LSMD varied in magnitude and strength by state, highlighting both the cosegregation of social adversity and poverty and the complexity of their interactions.

## 1. Introduction

Cutaneous melanoma is a significant public health concern with an estimated 76,380 cases diagnosed in 2016 and approximately 10,130 deaths as a result of this disease [1]. When diagnosed early, melanoma is associated with favorable survival rates (5-year overall survival for melanoma in situ and stage 1A melanomas is 99% and 97%, resp.) [2]. In contrast, the prognosis for advanced-stage melanoma is poor, with a 16% 5-year survival rate for patients with stage IV disease [2]. Hence, early and timely detection is critical to reducing mortality [3].

Despite the higher incidence of melanoma among non-Hispanic whites (NHW), Hispanics diagnosed with melanoma are 2.4 times [age adjusted odds ratio (OR), confidence interval (CI) 1.89–3.05] more likely to present with stage III disease [4] and 3.64 times more likely (CI 2.65–5.0) to have distant metastases than NHWs [3, 5–9]. Moreover, while the number of local stage or in situ melanomas has increased significantly among NHWs (indicative of improved screening efforts), the increasing melanoma incidence among Hispanic men living in California can be attributed to tumors thicker than 1.5 mm [10].

We do not know why Hispanics have worse melanoma outcomes. Late presentation of melanoma in Hispanics has been attributed to lack of awareness and knowledge [6], lower rates of self and physician performed skin examinations [7], differences in tumor biology [6], and socioeconomic forces. Primary care access may be particularly important for melanoma diagnosis and outcomes, given its importance to early diagnosis and the variability in health insurance coverage and healthcare access in this population. These factors may place Hispanic men at particular risk. US Hispanics are heterogeneous in many dimensions relevant to health outcomes [11] and display wide diversity in nativity, primary language, acculturation, education, and degree of social and spatial integration with non-Hispanic populations. Local lifestyle factors, access, and use of healthcare may shape the diagnosis, treatment, and progress of melanoma.

## 2. Conceptual Framework

The social ecological model (SEM) conceptually informs the design of this study. The SEM centers on the notion that spatial variations in health are due to characteristics of the environment, with ecological attributes of the (geographic) space affecting entire groups of people [12]. These attributes encompass a broad range of variables including language, culture, economic opportunity, healthcare resources, and utilization, as well as social integration with other racial and ethnic groups. Social determinants of late stage of diagnosis may operate at both the individual and neighborhood (census tract) levels and may be correlated. For example, immigrant Hispanics are more likely to use Spanish as their primary language, be in lower skilled occupations, have lower income, lack health insurance, live in immigrant enclaves, and live in an area with few healthcare resources [13, 14].

In this study, we (1) identify* places *at high risk for late stage diagnosis and (2) examine the contextual predictors of late stage diagnosis across three states (California, Texas, and Florida) in which just over half of the Hispanic population in the United States resides. We hypothesize that markers of small area ethnic concentration, residential segregation, tract economic disadvantage, and underservice by the healthcare system are each associated with a higher proportion of late stage diagnosis. We also hypothesize that the impact of these contextual correlates of late stage diagnosis varies among Hispanic men in each state because of variation in settlement patterns between states.

## 3. Material and Methods 

### 3.1. Study Design

We conducted a cross-sectional study using geocoded state cancer registry data from California, Texas, and Florida to examine the impact of neighborhood characteristics on melanoma stage at diagnosis in Hispanic men. We considered census tracts, which are relatively homogeneous with respect to their economic and living conditions, as a rough proxy for neighborhood [15, 16]. Census tracts have an average size of 4,000 inhabitants and generally cover a contiguous area [17]. A key advantage to census tract data is its relative stability, facilitating the comparison of data over time and space [17].

### 3.2. Data Source

The California, Texas, and Florida cancer registries collect information on incidence and clinically confirmed cancer stage at diagnosis. Each registry collects demographic, clinical, diagnostic (type of cancer, date of diagnosis, histology, and extent of disease), and treatment information (surgery, radiation, chemotherapy, and hormonal therapy).

### 3.3. Study Population

We limited data to histologically confirmed cutaneous melanoma [International Classification of Disease for Oncology, third edition site codes (C440–449) and histological codes (8720–8790)] reported to the registries during 1996 to 2012. We considered cases eligible for the study if registry data indicated Hispanic ethnicity, male gender, and age older than 18 years. Cases were identified as Hispanic, according to the North American Association of Central Cancer Registries Hispanic Identification Algorithm (NHIA). The algorithm uses the following variables to assign Hispanic ethnicity: Spanish/Hispanic origin, last name, maiden name, birthplace, race, and sex. We excluded 94, 43, and 81 cases due to missing census tract addresses in California, Texas, and Florida, respectively. This study was approved by the administrative committees and the respective Institutional Review Boards (IRB) for each state, Eastern Virginia Medical School, and Hampton University.

### 3.4. Cancer Cases

For each case, we collected information on race, ethnicity, age, summary stage, tumor thickness, histological subtype, treatment status, and census tract of residence at the time of diagnosis. We categorized histological subtype as superficial spreading, nodular, acral lentiginous, lentigo maligna, and other. We assigned census tracts using 2010 US Census geocodes.

The key dependent variable was stage at diagnosis: late stage versus early stage (Table 1). We defined late stage regional melanoma (direct extension only; regional, regional lymph nodes only; regional, direct extension and regional lymph nodes; regional, NOS), distant stage melanoma, and unknown stage. We included unknown stage in the late stage category since unknown staged melanoma has been shown to have comparable 5-year survival rates to regional stage melanoma [18]. We defined early stage as all melanoma recorded as “localized” and in situ.

#### 3.4.1. Neighborhood Measures

We explored several key area-based SES measures to capture different domains of socioeconomic position (poverty, income, and education), in relation to melanoma outcomes. Census tract socioeconomic data on percent of tract residents living below the federal poverty line, percent of tract residents, age 25 or older, possessing less than a high school education, and median household income were extracted from the 1990 and 2000 US Decennial Census and the American Community Survey (ACS), 2009–2012. To facilitate spatial modeling using a common set of geographic units, 1990 and 2000 Census data were obtained in 2010 boundaries [19]. To obtain census tract values for interdental years, we interpolated between 1990 and 2000 and 2000 and 2007 (the first available year for the ACS 5-year estimates), using linear interpolation for median household income and logistic interpolation for percent below poverty and percent less than high school education.

We categorized percent below poverty into high poverty (>20%), medium poverty (10–19.9%), and low poverty (<10%). Census tract education was classified into low, low-mid, mid, and high levels of educational attainment as 40–100%, 25–39.9%, 15–24.9%, and 0–14.9%, respectively. We defined cut points for these neighborhood socioeconomic measures based on the published literature [20]. We defined quintiles of median household income based on the census tract distribution across the three states. Segregation measures have been used in many studies of health and healthcare use in ethnic populations in two dimensions:* evenness* of residential distribution of a group to a comparison group or groups and* exposure* to other groups, or, its converse, isolation from other groups [21, 22].

We use the most commonly used measure of evenness, the index of dissimilarity. The index is measured as

(1) ∑HispanicTractHispanicCounty−Non-HispanicTractNon-HispanicCounty∗50,

where Group_Tract_ is the count of group members in the *i*th census tract and Group_County_ is the total population of that group in the county. The index of dissimilarity takes the value 0 if the shares of the groups compared residing in each tract within a county are identical and 100 if no members of the two groups coreside in the same tract. High values of the index are frequently interpreted as an indicator of residential avoidance and social distance between groups, which may indicate barriers to access to healthcare services [21, 23, 24]. The referent of the index of the index of dissimilarity is the larger unit of geography—in this case the county, with the county scores assigned to its component tracts.

We used three variables to capture the effects of ethnic isolation on late stage melanoma diagnosis: the proportion of tract residents who are Hispanic, the percentage of tract residents who are foreign born, and the proportion of tract residents who do not speak English well. In the context of studies of Hispanic health, high values of isolation measures are commonly interpreted as proxy indicators of continued strength of supportive community ties and cultural continuity, though they may also be interpreted as indices of isolation from acculturating influences [25, 26]. There is some disagreement among ethnic, immigrant, and language concentration as the most relevant indicator, and we investigate relationships to all three [25, 27, 28]. We chose to operationalize the isolation measures at the local (i.e., census tract) level, rather than using a regional average score, following the most common practice in Hispanic health studies, and because this is the more direct measure of the ethnic isolation construct. We defined cut points for each of these enclave variables using quartiles based on distribution across the three states.

We used two data sources to understand local availability of medical care resources: (1) Health Resources and Services Administration designated medically underserved areas (MUA) and (2) the Dartmouth Health Atlas Primary Care Service Area. MUAs are identified using the index of medical service (IMU) scale to designate an area as being medically underserved [29]. IMU is based on four variables: the ratio of primary care physicians (PCPs) per 1000 population; the infant mortality rate; the percent of population with income below the poverty level; and the percentage of population, age 65 or older. We assigned each cancer case an appropriate MUA value (0 = non-medically underserved area, 1 = medically underserved area, and 2 = medically underserved population).

We cross-walked census tract of residence with Dartmouth Health Atlas Primary Care Service Areas (PCSAs) to incorporate measures of adjusted rate of PCP intensity at the level of the PCSA [30]. We defined cut points based on distribution in the dataset as <57, 57–82, and >82 PCPs per 100,000 inhabitants per PCSA at the census tract level and assigned tracts a value according to their corresponding PCSA.

## 4. Statistical Analyses

We merged the case data with the census tract data by census tract and year. We summarize the sample characteristics and crude associations of each of the independent variables with melanoma stage at diagnosis (early versus late) in Table 2. We tested for differences in these variables between early and late stage melanoma cases using Student's* t*-test or the Wilcoxon-Mann-Whitney test for continuous variables and Chi-square test or Fisher's exact test for categorical covariates.

To model the impact of individual-level covariates, neighborhood socioeconomic measures, and Hispanic enclave measures on late stage cancer melanoma diagnosis in Hispanic men in California, Texas, and Florida, we adopted a hierarchical multilevel logistic regression model as follows: the modeling framework was as follows.

Let *y*_*ij*_ be an indicator variable taking a value of zero if patient *i* in area *j* was diagnosed early and 1 otherwise. We model

(2) yij~Bernoullipij,logit⁡p=β0+β1x1+⋯+βqxq+uj,

where *u*_*j*_ is a normally distributed random area effect with mean 0 and variance *σ*_*u*_^2^. The covariates *x*_1_,…, *x*_*q*_ included age, census tract median household income, education, poverty, medically underserved area, adjusted rate of allocated PCPs, and Hispanic enclave variables (i.e., the percentage of tract's Hispanics who were fluent in English, Hispanic, and foreign born). The multilevel logistic model was fit separately for each state using the Glimmer procedure in SAS version 9.4 (SAS Institute, Cary, NC).

We initially attempted to fit this model with census tract level random effects but found that model fitting was unstable due to the scarcity of cases among Hispanic men in many of the census tracts. We accordingly adopted the following hybrid approach. If the number of Hispanic male melanoma cases was ≥5, we retained the census tract as the unit of geography for the random effects specification. If the number of cases was <5, we aggregated these cases into the census county subdivision in which the census tract was located. The census tract/county division crosswalk was obtained from MABLE/Geocorr [31]. This permitted us to obtain estimates for census tracts with sufficient cases to allow for stable estimation. This algorithm resulted in 70, 846, and 1006 census tracts and 2819, 4170, and 3342 county subdivisions in California, Texas, and Florida, respectively. Neighborhood covariate values including the ethnic isolation measures for all cases were still assigned based on tract residence. The dissimilarity index score was in all cases calculated for distribution to tracts within counties and the resulting value assigned to the subcounty units.

Interactions terms between education, poverty, and Hispanic enclave measures were systematically considered in the multilevel model and retained based on their significance. The effects of the predictors on the outcome are given as odds ratios and 95% confidence intervals. The predicted estimates from the multilevel models were used in Arc Geographic Information System (GIS) to visualize geographic patterns of late stage melanoma. All *P* values less than 0.05 were considered statistically significant.

To explore state differences in the overall prevalence of late stage melanoma and covariate effects, we also fit a pooled model over all states and tested for significant interactions between state and the final set of covariates from the state-specific models.

## 5. Results

We identified 12,493 melanoma cases in Hispanic men residing in California, Texas, or Florida for 1996–2012. The demographic and clinical characteristics are presented in Table 2. The Hispanic populations and communities of the three states differ markedly, particularly between Texas and California versus Florida. Hispanics in Florida are older and more likely to be immigrants, but they also have on average higher levels of education.

Table 3 shows a correlation matrix for selected tract-level variables for each state using 2006–2011 American Community Survey data, weighted for the Hispanic population of each tract. In Florida neither ethnic nor immigrant concentration in census tracts is associated with economic disadvantage or a lower ratio of PCPs to population, in contrast to both Texas and California. In California and Florida, ethnic concentration (percent Hispanic) is strongly associated with immigrant enclaves (percent immigrant). By contrast, in Texas, tract-level ethnic concentration frequently occurs even in the US-born Hispanic population, especially near the Mexican border where US-born Hispanics comprise large majorities.

**Figure 1:**
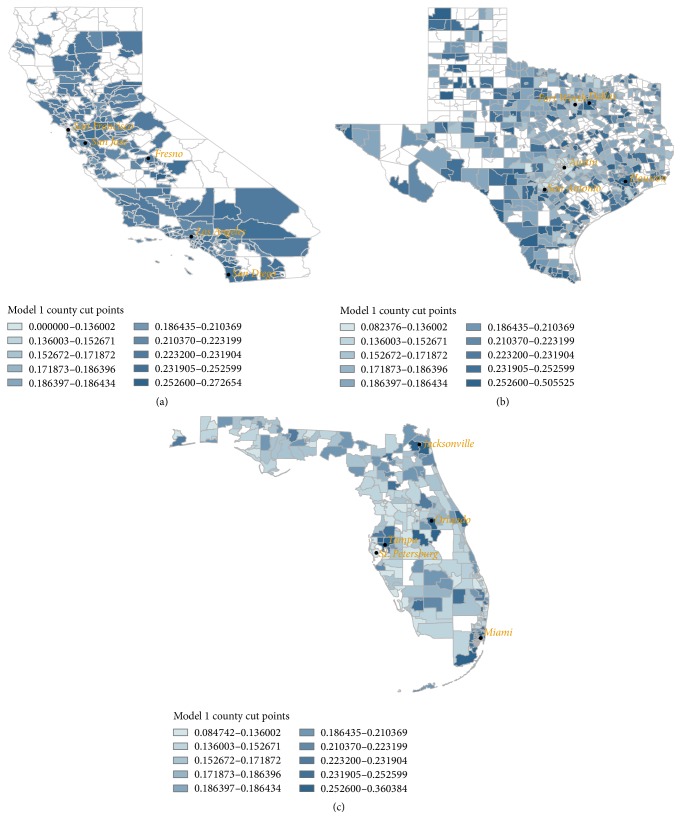
Spatial distribution of late stage diagnosis. ((a)–(c)) Each map depicts the prevalence of late stage melanoma diagnosis in Hispanic men in California, Texas, and Florida, respectively. Maps were generated using the Geographic Information System (GIS). All *P* values less than 0.05 were considered statistically significant.

In Figure 1, state-specific maps display the spatial distribution of late stage diagnosis. States differed markedly in the prevalence of late stage disease. California had the highest rates of late diagnosis with late stage cases clustering in the southern corner of the state. In Texas, late diagnosis tended to concentrate in the border areas; in Florida, rates of late stage diagnosis were greatest along the southeast coastline.

Table 4 displays the significant contrasts in neighborhood characteristics by stage at diagnosis. Across the three states, a greater proportion of late stage versus early stage cases resided in areas characterized by higher levels of poverty, lower levels of educational attainment, and lower median household income. A greater percentage of late stage cases was found in census tracts containing a higher density of Hispanics, immigrants, and individuals with limited knowledge of English. A greater proportion of late stage cases lived in medically underserved and PCP shortage areas.

The minimally adjusted (Model 1) and fully adjusted (Model 2) odds ratios (with 95% confidence intervals) for late stage diagnosis are shown in Table 5. In California, late stage cases remained significantly more likely than early stage cases to reside in areas composed of Hispanics (being the majority) [OR (95% CI): 2.4 (1.5–3.7) for >60% Hispanic], immigrants [OR (95% CI): 2.1 (1.0–4.5) for >65% immigrants], and those with fewer PCPs [OR (95% CI): 1.4 (1.0–1.9) for <57 PCPs]. In Texas, late stage melanoma was associated with higher odds of residence in areas of socioeconomic deprivation [OR (95% CI): 2.6 (1.7–3.9) lowest levels of education attainment and 1.8 (1.3–2.4) for increased poverty], higher proportion of immigrants [OR (95% CI): 2.1 (1.1–3.9) for >65% immigrants], and low PCP supply [OR (CI): 1.6 (1.2–2.1) for <57 PCPs]. In Florida, living in areas of low education attainment [OR (95% CI): 1.8 (1.1–2.8) lowest levels of education attainment], high poverty [OR (95% CI): 1.8 (1.3–2.5)], and a high percentage of residents identifying as Hispanic [OR (95% CI): 1.9 (1.4–2.5) for >60% Hispanic] was significantly associated with late stage melanoma diagnosis. Across all three states, the index of dissimilarity did not impact the odds of late stage melanoma diagnosis.

## 6. Discussion

We used state registry population-based data to investigate the relationship between neighborhood characteristics and melanoma stage at diagnosis among Hispanic men in California, Texas, and Florida. Our analysis, including approximately 12,493 patient cases, reveals five major findings. First, residence in census tracts with high immigrant density (California and Texas) and a high composition of Hispanics (California and Florida) was a significant predictor of late stage melanoma diagnosis in fully adjusted models. Second, the strength of the association between SES measures (poverty and education) and stage of diagnosis was attenuated in multivariate models when enclaves and health resource related factors were taken into account. Third, we found an increased likelihood of late stage diagnosis in areas with low PCP density in California and Texas. Fourth, the probability of late stage diagnosis concentrates in specific regions along the US/Mexico borders, in south central California, and in the southeast coast of Florida. Lastly, in Texas, Hispanic men, ages 18–34 and 35–49, were at increased risk for late stage diagnosis compared to men 65 years or older.

While our results suggest that the spatial aggregation of immigrants and Hispanics into neighborhood enclaves adversely affects melanoma outcomes, they also imply that the attributes of immigrant and Hispanic enclaves vary by state of settlement. For example, Hispanic clustering is correlated with immigrant status more strongly in California and Florida than in Texas. While California and Texas show an association of Hispanic/immigrant and SES measures of disadvantage, Florida does not. Prior studies have found that segregation is an important determinant of racial and ethnic health disparities because it effectively measures social distance [23, 32]. However, we did not find an independent effect of model of late stage melanoma diagnosis.

Numerous studies have reported the association of late stage cancer diagnosis and residence in census tracts with high proportions of Hispanics and immigrants [27, 33–35]. Gomez et al. found that foreign-born Hispanic women living in low-SES high-enclave neighborhoods were at greater risk of being diagnosed with late stage cervical cancer than those in high-SES low-enclave neighborhoods [34]. Reyes-Ortiz et al. found that while Hispanics living in densely populated neighborhoods may benefit from lower cancer incidence, they were burdened by late stage diagnosis and larger tumors of the breast, cervix, and colon [33]. Although immigrant and Hispanic enclaves have been shown to serve as sources of social support [27], they may also negatively influence health via barriers to assimilation, linguistic isolation, lack of access to medical care, and health information and by reinforcing health-related behavioral norms. Residence in enclaves may also reinforce negative skin-related health behaviors and foster false perceptions regarding skin cancer risks. Studies show significantly lower rates of skin self-examinations [36–41] and physician-assisted skin examinations [41–43] among Hispanics compared to NHWs. Among Hispanics, factors associated with skin self-examinations and receipt of physician-assisted skin examinations include greater US acculturation, older age, an increased number of melanoma risk factors, physician recommendations [44, 45], country of origin [46], elevated levels of skin cancer knowledge, and heightened awareness of perceived skin cancer risks.

The greater likelihood of late stage melanoma diagnosis for Hispanics in lower income tracts has been previously reported. A recent investigation found that the association of thicker tumors (>2 mm) and low SES was stronger among Hispanic men (relative risk [RR] 2.18; CI 1.73–2.74) and women (RR 1.98; 1.55–2.51) compared to their NHW counterparts, suggesting Hispanics may be disproportionately burdened by barriers to screening and care due to poverty [10]. Hu et al. found that, for every 1% increase in the population living below the federal poverty level, the odds among Hispanics of living in a high risk cluster increased 2% [47]. Our findings from adjusted analyses point to a more complex relationship between socioeconomic indicators and late stage diagnosis, particularly in California where the influence of poverty and education was attenuated after controlling for health access and enclave measures. Prior studies have shown that educational and income gradients may behave differently for Hispanics across various health outcomes. Goldman et al. found a weak association between education and select health behaviors and outcomes for foreign- and US-born Mexicans [48]. Perhaps in California, a state in which immigrant and Hispanic status of neighborhoods are highly correlated, SES may be of less relevance than cultural factors (cultural norms, reinforced health-related skin behavior), suggesting that the health advantages of upward economic mobility are counteracted by strong cultural perceptions and norms.

We found that cases residing in PCSAs with lower PCP availability in California and Texas, but not Florida, had higher odds of late stage diagnosis. PCSAs, small geographic units defined by utilization data, approximate self-contained markets for ambulatory primary care services [19]. Previous studies do not agree on the influence of health professional shortages on health outcomes [49–51]. In our study, poor PCP supply in Florida did not independently predict late stage diagnosis. As demonstrated by Allen et al., socioeconomic and cultural determinants may outweigh the importance of physician supply [52]. Alternatively, deficiencies in physician supply may be buffered by services provided by physician extenders, but additional studies are required to confirm this. Notably, the association of lack of surgical intervention with late stage diagnosis was most profound in California. Additional analyses (data not shown) revealed that this finding was unrelated to individual primary payer status. The impact of PCSA on receipt of surgical treatment is unclear, but it may signal deficiencies in access to oncologic services and inefficient care coordination.

While studies have explored primary and dermatologic care access and melanoma outcomes in NHWs [39], we are unaware of published studies on the association of healthcare access and melanoma burden in Hispanics. This is especially important for melanoma, which is amenable to screening by primary practitioners and particularly relevant for Hispanics who experience substantial obstacles to basic healthcare. More than one-quarter of Hispanic adults do not consistently visit a PCP and Hispanics are twice as likely as non-Hispanic blacks and three times as likely as NHWs to have no regular healthcare provider [40]. Further, undocumented immigrants are ineligible for the Federal Marketplace [41] of Medicaid [42] and, if Medicaid eligible, may face a 5-year waiting period [41]. Contextual variables that impact access to care and treatment also impact access to screening and screening use [43], a behavior with respect to melanoma that Hispanics infrequently engage in [31]. Further investigation is warranted of how immigration status, insurance status, and healthcare access influence melanoma outcomes in the Hispanic population.

We detected a robust association of late stage diagnosis with primary melanoma of the lower limbs across all three states. Numerous studies have reported the increased prevalence of lower extremity melanoma among minorities, including Hispanics [4, 5]. Unfortunately, the nature of cancer registry data hindered our precise characterization of tumor location and histological subtype. Melanoma of the lower extremity may be less conspicuous and therefore remain undetected by both patients and their providers. Alternatively, melanoma of the lower extremities may signal the presence of distinct etiological pathways and risk factors for Hispanics.

## 7. Strengths and Limitations

The strengths of this study include its large population-based sample, its inclusion of states which together account for ~55% of US Hispanics, and the inclusion of numerous area-based measures of SES, healthcare resources, and Hispanic enclaves. However, we note several important limitations. Similar to other cancer registry-based investigations, we were unable to examine potential confounding individual-level factors such as health insurance status and comorbidities. We were also unable to characterize the phenotypic profile of our patient cases; this is especially important for melanoma, as skin and hair color are established risk factors for melanoma development. Similarly, measures of ethnic environments were based on census measures of population prevalence, rather than more fine-grained measures of ethnic relations and interaction. Misclassification of Hispanic ethnicity is a third possible limitation; however, the North American Association of Cancer Registries Hispanic Identification Algorithm utilized to identify our patient cases minimizes such misclassification [53]. This population could potentially be overlooked as PCSAs are derived from Medicare claims. Despite this limitation, prior studies have shown the applicability of PCSAs to Medicaid and private insurance, implicating their utility in this study [38].

## 8. Conclusion

In conclusion, we found that the risk of late stage melanoma diagnosis among Hispanic men varied by neighborhood socioeconomic factors, residence in ethnic and immigrant enclaves, and residence in low primary care resource settings. The influence of these exposures varied in magnitude and strength by state, highlighting both the cosegregation of social adversity and poverty, and the complexity of their interactions. Further scrutiny is warranted into the role of cultural factors, nativity, insurance status, and healthcare access in melanoma screening and diagnosis among Hispanics. The main objective of our paper was to show the spatial variation in late stage melanoma diagnosis across multiple domains related to SES, ethnic concentration, and healthcare resource allocation. However other contextual measures, such as transportation and housing, may also be related to delayed diagnosis, and variables in these domains are likely correlated with the SES measures used in our analysis. Future research investigating how factors such as housing, transportation, and occupation (and other contextual measures) drive the observed associations between socioeconomic deprivation, ethnic concentration, healthcare resource allocation, and late stage melanoma diagnosis is warranted.

## Supplementary Material

Supplemental Figure 1. Spatial distribution of contextual characteristics in California, Texas, and Florida.

## Figures and Tables

**Table 1:** Distribution of patient cases by summary stage, California, Texas, and Florida, 1996–2012.

Summary stage at diagnosis	California		Texas		Florida		Category
	Frequency	%	Frequency	%	Frequency	%	
*In situ*	875	29.44	2123	42.35	1925	42.64	*Early*
*Localized*	1385	46.60	1804	35.99	1785	39.53	

*Regional by direct extension*	76	2.56	67	1.34	53	1.17	*Late*
*Regional by lymph nodes*	195	6.56	142	2.83	141	3.12	
*Regional by direct extension and lymph nodes*	47	1.58	36	0.72	28	0.62	
*Regional, NOS*	26	0.87	6	0.12	11	0.24	
*Remote*	225	7.57	189	3.77	153	3.39	
*Unknown or not specified*	143	4.81	646	12.89	419	9.28	

**Table 2:** Demographic and clinical characteristics of melanoma cases in Hispanic men by stage, California, Texas, and Florida, 1996–2012.

	California			Texas			Florida		
	Early (*N* = 2260)	*P* value	Late (*N* = 712)	Early (*N* = 3927)	*P*-value	Late (*N* = 1086)	Early (*N* = 3710)	*P* value	Late (*N* = 805)
	Number (%)		Number (%)	Number (%)		Number (%)	Number (%)		Number (%)
*Demographic variables*									
*Age at diagnosis (mean ± SD)*	59.3 ± 17.2	0.0175	57.5 ± 18	60.6 ± 15.1	0.1554	59.9 ± 16.2	65.2 ± 26.4	0.0286	63.1 ± 16.4
*Age group*									
18–34	182 (8.1)	0.1876	68 (9.6)	207 (5.3)	0.0153	75 (6.9)	116 (3.1)	0.0282	38 (4.7)
35–49	480 (21.2)		168 (23.6)	716 (18.2)		223 (20.5)	502 (13.5)		122 (15.2)
50–64	630 (27.9)		2192 (27.0)	1338 (34.1)		327 (30.1)	1002 (27)		226 (28.1)
65 and older	954 (42.2)		274 (38.5)	1667 (42.5)		461 (42.5)	2081 (56.1)		414 (51.4)
Missing	14 (0.6)		10 (1.4)	0 (0.0)		0 (0.0)	9 (0.2)		5 (0.6)
*Race/ethnicity*									
White	2112 (93.5)	<0.0001	702 (98.6)	2515 (64.0)	<0.0001	820 (75.5)	1297 (35)	<0.0001	514 (63.9)
Black	0 (0.0)		2 (0.3)	68 (1.7)		79 (7.3)	121 (3.3)		104 (12.9)
Other	2 (0.1)		0 (0.0)	100 (2.6)		56 (5.2)	197 (5.3)		60 (7.5)
Unknown	146 (6.5)		8 (1.1)	1244 (31.7)		131 (12.1)	2095 (56.5)		127 (15.8)
*Primary payer at diagnosis*									
No insurance	95 (4.2)	<0.0001	53 (7.4)	53 (1.4)	<0.0001	51 (4.7)	110 (3.0)	<0.0001	63 (7.8)
Private insurance	1236 (54.7)		298 (41.9)	258 (6.6)		106 (9.8)	1291 (34.8)		262 (32.6)
Medicaid	117 (5.2)		117 (16.4)	8 (0.2)		19 (1.8)	50 (1.4)		43 (5.3)
Medicare	503 (22.3)		172 (24.2)	205 (5.2)		113 (10.4)	1218 (32.8)		272 (33.8)
Other	27 (1.2)		15 (2.1)	55 (1.4)		7 (0.6)	43 (1.2)		11 (1.4)
Unknown	282 (12.5)		57 (8.0)	1800 (45.8)		317 (29.2)	998 (26.9)		154 (19.1)
Missing	0 (0.0)		0 (0.0)	1548 (39.4)		473 (43.6)	0 (0.0)		0 (0.0)

*Clinical characteristics*									
*Primary site*									
Head and neck	833 (37)	<0.0001	119 (16.7)	1383 (35.3)	<0.0001	245 (22.6)	1190 (32)	<0.0001	161 (20.1)
Trunk	660 (29.2)		126 (17.7)	1194 (30.4)		253 (23.3)	1257 (33.9)		198 (24.6)
Upper extremity	384 (17.0)		93 (13.1)	888 (22.6)		162 (14.9)	865 (23.3)		108 (13.4)
Lower extremity	370 (16.4)		195 (27.4)	385 (9.8)		215 (19.8)	371 (10)		146 (18.1)
Overlap	1 (0.0)		0 (0.0)	6 (0.2)		4 (0.4)	2 (0.1)		1 (0.1)
Unknown	12 (0.5)		179 (25.1)	71 (1.8)		207 (19.1)	25 (0.7)		191 (23.7)
*Tumor thickness*									
T0	11 (0.5)	<0.0001	68 (9.6)	17 (0.4)	<0.0001	13 (1.2)	19 (0.5)	<0.0001	22 (2.7)
T1	794 (35.1)		59 (8.3)	630 (16.0)		113 (10.4)	776 (20.9)		82 (10.2)
T2	274 (12.1)		65 (9.1)	40 (1.0)		13 (1.2)	115 (3.1)		21 (2.6)
T3	157 (7.0)		98 (13.8)	17 (0.4)		13 (1.2)	69 (1.9)		30 (3.7)
T4	76 (3.4)		128 (18.0)	18 (0.5)		19 (1.8)	40 (1.1)		42 (5.2)
9.80 mm or larger	5 (0.2)		33 (4.6)	4 (0.1)		8 (0.7)	7 (0.2)		20 (2.5)
Unknown	943 (41.7)		261 (36.7)	2468 (62.9)		716 (65.9)	1496 (40.3)		263 (32.7)
Missing	0 (0.0)		0 (0.0)	733 (18.7)		191 (17.6)	1188 (32)		325 (40.4)
*Presence of ulceration*									
No ulceration present	1201 (53.1)	<0.0001	172 (24.2)	754 (19.2)	<0.0001	48 (4.4)	1470 (39.6)	<0.0001	109 (13.5)
Ulceration present	137 (6.1)		141 (19.8)	48 (1.2)		22 (3.0)	107 (2.9)		83 (10.3)
Not collected, unknown, and missing	922 (40.8)		399 (56)	3125 (79.6)		1005 (92.6)	2133 (57.5)		613 (76.2)
*Lymph node status*									
No lymph node metastases	1368 (60.5)		116 (16.3)	947 (24.1)		48 (4.4)	2381 (64.2)		115 (14.3)
Clinically positive lymph nodes and/or in-transit metastasis	2 (0.1)	<0.0001	184 (25.9)	—	<0.0001	59 (5.4)	1 (0.0)	<0.0001	119 (14.8)
Information not collected, unknown, and missing	890 (39.4)		412 (57.9)	2980 (75.9)		979 (90.2)	1328 (35.8)		571 (71)
*Reason for no surgery*									
Surgery was performed	2138 (94.6)	<0.0001	483 (67.8)	2320 (59.1)	<0.0001	598 (55.1)	3335 (89.9)	<0.0001	577 (71.7)
Surgery not performed	83 (3.7)		203 (28.5)	67 (1.7)		118 (10.9)	344 (9.3)		179 (22.2)
Recommended, not given, refused, unknown if performed, patient died prior to surgery, or surgery contraindicate due to patient risk factors	30 (1.3)		10 (1.4)	16 (0.4)		21 (2.0)	15 (0.5)		8 (1.1)
Unknown, diagnosed at autopsy, or death certificate only	9 (0.4)		8 (1.1)	822 (20.9)		240 (22.1)	16 (0.4)		41 (5.1)
Missing	0 (0.0)		0 (0.0)	702 (17.9)		109 (10)	—		—
*Diagnostic confirmation*									
Histology, including hematology	2260 (100.0)	<0.0001	683 (95.9)	3923 (99.9)	<0.0001	993 (91.4)	3704 (99.8)	<0.0001	762 (94.7)
Other^*∗∗*^ and unknown	0 (0.0)		29 (4.1)	4 (0.1)		93 (8.6)	6 (0.2)		43 (5.3)
*Histologic type *									
Melanoma NOS	1199 (53.1)	<0.0001	481 (67.6)	2432 (61.9)	<0.0001	722 (66.5)	2899 (77.9)	<0.0001	646 (80.3)
Nodular	125 (5.5)		128 (18.0)	89 (2.3)		66 (6.1)	97 (2.6)		66 (8.2)
Lentigo maligna	371 (16.4)		2 (0.3)	544 (13.9)		88 (8.1)	334 (9.0)		7 (0.9)
Superficial spreading	471 (20.8)		53 (7.4)	753 (19.2)		119 (11)	269 (7.3)		22 (2.7)
Acral lentiginous	94 (4.2)		48 (6.7)	56 (1.4)		53 (4.9)	50 (1.4)		27 (3.4)
Other^*∗*^	—		—	53 (1.4)		38 (3.6)	71 (2.0)		37 (4.6)

^*∗∗*^Other forms of diagnostic confirmation: cytology, microscopically confirmed, but type of specimen unknown, positive lab test or marker study, direct observation, radiography, clinical, and other.   ^*∗*^Other histologic types with corresponding histology codes: balloon cell melanoma (8722); malignant melanoma regressing (8723); amelanotic melanoma (8730); melanoma in junctional nevus (8740); melanoma in precancerous melanosis (8741); desmoplastic melanoma (8745); mucosal lentiginous melanoma (8746); melan giant pig nevus (8761); epithelioid/spindle cell melanoma (8770); epithelial cell melanoma (8771); spindle cell melanoma (8772); and blue nevus malignant (8780); *P* values are applied within each state to compare early and late stage disease.

**Table 3:** Correlation matrix for tract level contextual variables by state^*∗*^.

State		% of a tract's residents who are Hispanic	% of a tract's Hispanic residents who are foreign-born or born outside of the 50 states/DC	% of tract Hispanics who say they speak English not well or not at all	% of tract Hispanics living below the FPL^*∗∗*^
California	% of a tract's Hispanic residents who are foreign-born or born outside of the 50 states/DC	0.50			
	% of tract Hispanics who say they speak English not well or not at all	0.63	0.83		
	% of tract Hispanics living below the FPL	0.38	0.33	0.49	
	Allocated primary care physicians that are clinically active (PCSA)/100,000 population	−0.38	0.06	−0.10	−0.18

Texas	% of a tract's Hispanic residents who are foreign-born or born outside of the 50 states/DC	0.07			
	% of tract Hispanics who say they speak English not well or not at all	0.34	0.82		
	% of tract Hispanics living below the FPL	0.42	0.23	0.45	
	Allocated primary care physicians that are clinically active (PCSA)/100,000 population	−0.39	−0.07	−0.14	−0.12

Florida	% of a tract's Hispanic residents who are foreign-born or born outside of the 50 states/DC	0.64			
	% of tract Hispanics who say they speak English not well or not at all	0.52	0.66		
	% of tract Hispanics living below the FPL	−0.02	0.01	0.35	
	Allocated primary care physicians that are clinically active (PCSA)/100,000 population	0.34	0.38	0.21	−0.06

^*∗*^Based on data from the American Community Survey; ^*∗∗*^FPL: federal poverty level.

**Table 4:** Contextual characteristics of Hispanic men diagnosed with melanoma by stage in California, Texas, and Florida, 1996–2012.

	California			Texas			Florida		
Contextual variables	Early (*N* = 2260)	*P* value	Late (*N* = 712)	Early (*N* = 3927)	*P* value	Late (*N* = 1086)	Early (*N* = 3710)	*P* value	Late (*N* = 805)
	Number (%)		Number (%)	Number (%)		Number (%)	Number (%)		Number (%)

*Education (% less than HS* ^*∗*^)									
0–14.9%	941 (41.6)	<0.0001	181 (25.4)	2302 (58.6)	<0.0001	376 (34.6)	2210 (59.6)	<0.0001	323 (40.1)
15–24.9%	382 (16.9)		118 (16.6)	752 (19.2)		232 (21.4)	749 (20.2)		184 (22.9)
25–39.9%	415 (18.4)		156 (21.9)	502 (12.8)		214 (19.7)	426 (11.5)		168 (20.9)
40–100%	435 (19.3)		237 (33.3)	315 (8.0)		241 (22.2)	166 (4.5)		95 (11.8)
Missing	87 (3.9)		20 (2.8)	56 (1.4)		23 (2.1)	159 (4.3)		35 (4.4)
*% of tract Hispanics living below the FPL*									
<10%	1072 (47.4)	<0.0001	242 (34.0)	2109 (53.7)	<0.0001	349 (32.1)	2076 (56.0)	<0.0001	315 (39.1)
10–19.9%	643 (28.5)		227 (31.9)	1087 (27.7)		309 (28.5)	1085 (29.3)		267 (33.2)
≥20%	459 (20.3)		220 (30.9)	689 (17.6)		408 (37.6)	386 (10.4)		190 (23.6)
Missing	86 (3.8)		23 (3.2)	42 (1.1)		20 (1.8)	163 (4.4)		33 (4.1)
*Median household income*									
7,594–$42,064	787 (34.8)	<0.0001	340 (47.8)	853 (21.7)	<0.0001	424 (39.0)	782 (21.1)	<0.0001	259 (32.2)
42,065–$52,506	490 (21.7)		165 (23.2)	684 (17.4)		224 (20.6)	619 (17.4)		132 (16.4)
52,507–$65,310	391 (17.3)		101 (14.2)	678 (17.3)		162 (14.9)	670 (18.1)		126 (15.7)
65,311–$87,847	303 (13.4)		50 (7.0)	810 (20.6)		143 (13.2)	648 (17.5)		111 (13.8)
87,848–$244,876	213 (9.4)		36 (5.1)	872 (22.2)		116 (10.7)	837 (22.6)		144 (17.9)
Missing	76 (3.4)		20 (2.8)	30 (0.8)		17 (1.6)	154 (4.2)		33 (4.1)

Ethnic concentration measures	Early (*N* = 2260)	*P* value	Late (*N* = 487)	Early (*N* = 3928)	*P* value	Late (*N* = 896)	Early (*N* = 3710)	*P* value	Late (*N* = 652)
	Number (%)		Number (%)	Number (%)		Number (%)	Number (%)		Number (%)

*% of tract Hispanics who say they speak English not well or not at all*									
0–3	304 (13.5)	<0.0001	54 (7.58)	870 (22.2)	<0.0001	148 (13.6)	803 (21.6)	<0.0001	118 (14.7)
>3–10	467 (20.7)		97 (13.6)	1203 (30.6)		257 (23.7)	684 (18.4)		98 (12.2)
>10–25	821 (36.3)		276 (38.8)	1276 (32.5)		417 (38.4)	1171 (31.6)		262 (32.6)
>25	592 (26.2)		265 (37.2)	545 (13.9)		246 (22.7)	851 (22.9)		295 (36.7)
Missing	76 (3.4)		20 (2.8)	33 (0.8)		18 (1.7)	201 (5.4)		32 (4.0)
*% of a tract's Hispanic residents who are foreign-born or born outside of the 50 states/DC*									
<25	483 (21.4)	<0.0001	102 (14.3)	1875 (47.8)	<0.0001	430 (39.6)	201 (5.4)	<0.0001	29 (3.6)
>25–40	788 (34.9)		224 (31.5)	1232 (31.4)		337 (31.0)	432 (11.6)		74 (9.2)
>40–65	882 (39.0)		350 (49.2)	74 (19.0)		281 (25.9)	1680 (45.3)		302 (37.5)
>65	31 (1.4)		16 (2.3)	41 (1.0)		20 (1.8)	1196 (32.2)		368 (45.7)
Missing	76 (3.4)		20 (2.8)	32 (0.8)		18.0 (1.7)	201 (5.4)		32 (4.0)
*% of a tract's residents who are Hispanic*									
0–20	763 (33.8)	<0.0001	134 (18.8)	2103 (53.6)	<0.0001	393 (36.2)	2270 (61.2)	<0.0001	346 (43.0)
>20–40	514 (22.7)		129 (18.1)	866 (22.1)		210 (19.3)	542 (14.6)		144 (17.9)
>40–60	349 (15.4)		134 (18.8)	383 (9.8)		131 (12.1)	277 (7.5)		72 (8.9)
>60	559 (24.7)		296 (41.6)	546 (13.9)		335 (30.9)	472 (12.7)		215 (26.7)
Missing	75 (3.3)		19 (2.8)	29 (0.7)		17 (1.6)	149 (4.0)		28 (3.5)

Healthcare resource allocation	Early (*N* = 2260)	*P* value	Late (*N* = 487)	Early (*N* = 3928)	*P* value	Late (*N* = 896)	Early (*N* = 3710)	*P* value	Late (*N* = 652)
	Number (%)		Number (%)	Number (%)		Number (%)	Number (%)		Number (%)

*Allocated primary care physicians that are clinically active (PCSA)/100,000 population*									
<57	411 (18.2)	<0.0001	172 (24.2)	1207 (30.7)	<0.0001	482 (44.4)	601 (16.2)	0.1378	126 (15.6)
57–82	998 (44.2)		356 (50.0)	2013 (51.3)		473 (43.6)	2147 (57.9)		498 (61.9)
>82	776 (34.3)		165 (23.2)	679 (17.3)		115 (10.6)	814 (21.9)		157 (19.5)
Missing	75 (3.3)		19 (2.7)	28 (0.7)		16 (1.5)	148 (4.0)		24 (3.0)
*Medically underserved area*									
Not a MUA/P	1718 (76.0)	<0.0001	19 (2.7)	2483 (63.2)	<0.0001	572 (52.7)	1886 (50.8)	<0.0001	367 (45.6)
Med. unders. area	332 (14.7)		494 (69.4)	1371 (34.9)		467 (43.0)	1184 (31.9)		201 (25.0)
Med. unders. pop.	135 (6.0)		143 (20.1)	45 (1.2)		31 (2.9)	496 (13.4)		213 (26.5)
Missing	75 (3.3)		56 (7.9)	28 (0.7)		16 (1.5)	144 (3.9)		24 (3.0)

^*∗*^HSS = high school.

**Table 5:** Odds ratios for late melanoma diagnosis and census tract contextual variables among Hispanic men, California, Texas, and Florida, 1996–2012.

	California						Texas						Florida					
	Minimally unadjusted (*N* = 2,920)			Fully adjusted (*N* = 2,870)			Minimally unadjusted (*N* = 5,046)			Fully adjusted (*N* = 4,992)			Minimally unadjusted (*N* = 4,386)			Fully adjusted (*N* = 4,280)		
	Odds ratio estimates	95% confidence limits		Odds ratio estimates	95% confidence limits		Odds ratio estimates	95% confidence limits		Odds ratio estimates	95% confidence limits		Odds ratio estimates	95% confidence limits		Odds ratio estimates	95% confidence limits	
*Age group*																		
18–34	1.3	0.9	1.8	1.3	0.9	1.8	1.5	1.1	2.0	1.6	1.2	2.2	1.5	1.0	2.2	1.6	1.0	2.3
35–49	1.3	1.0	1.6	1.2	1.0	1.5	1.2	1.0	1.5	1.3	1.1	1.6	1.2	0.9	1.5	1.2	1.0	1.6
50–64	1.1	0.9	1.3	1.0	0.8	1.2	0.9	0.8	1.1	1.0	0.9	1.2	1.1	0.9	1.3	1.1	0.9	1.3
≥65	—	—	—	—	—	—	—	—	—	—	—	—	—	—	—	—	—	—
*Education (% less than HS)*																		
0–14.9%	—	—	—	—	—	—	—	—	—	—	—	—	—	—	—	—	—	—
15–24.9%	1.6	1.2	2.1	1.3	0.8	1.5	1.9	1.5	2.3	1.6	1.3	2.1	1.7	1.3	2.1	1.4	1.1	1.8
25–39.9%	1.9	1.5	2.4	1.2	0.6	1.3	2.5	2.0	3.1	1.8	1.3	2.4	2.5	1.9	3.1	1.7	1.3	2.4
40–100%	2.7	2.1	3.4	1.0	0.6	1.6	4.2	3.3	5.2	2.5	1.7	3.8	3.4	2.4	4.7	1.8	1.2	2.8
*Percent of tract Hispanics living below the FPL*																		
<10%	—	—	—	—	—	—	—	—	—	—	—	—	—	—	—	—	—	—
10–19.9%	1.6	1.3	1.9	1.0	0.8	1.3	1.7	1.4	2.0	1.3	1.0	1.6	1.6	1.3	1.9	1.2	0.9	1.5
≥20%	2.0	1.6	2.5	1.1	0.8	1.5	3.2	2.7	3.9	1.7	1.3	2.3	2.9	2.2	3.6	1.8	1.3	2.4
*% of tract Hispanics who say they speak English not well or not at all*																		
0–3	—	—	—	—	—	—	—	—	—	—	—	—	—	—	—	—	—	—
>3–10	1.1	0.8	1.6	0.9	0.6	1.3	1.3	1.0	1.6	1.0	0.8	1.3	1.0	0.7	1.3	0.9	0.7	1.2
>10–25	1.8	1.3	2.5	1.0	0.7	1.6	1.7	1.4	2.1	0.9	0.7	1.2	1.4	1.1	1.8	1.0	0.8	1.4
>25	2.3	1.7	3.3	0.9	0.6	1.6	2.3	1.8	2.9	0.7	0.5	1.0	2.0	1.5	2.6	0.8	0.6	1.1
*% of a tract's residents who are Hispanic*																		
0–20	—	—	—	—	—	—	—	—	—	—	—	—	—	—	—	—	—	—
>20–40	1.5	1.1	1.9	1.3	0.9	1.8	1.4	1.1	1.7	1.0	0.8	1.3	1.6	1.3	2.1	1.4	1.1	1.8
>40–60	2.2	1.7	2.9	1.8	1.3	2.7	1.8	1.4	2.3	1.0	0.7	1.3	1.6	1.2	2.3	1.4	1.0	2.1
>60	3.0	2.3	3.8	2.4	1.6	3.8	3.2	2.6	4.0	1.3	0.9	1.8	2.8	2.2	3.6	2.2	1.5	3.2
*% of a tract's Hispanic residents who are foreign-born or born outside of the 50 states/DC*																		
<25	—	—	—	—	—	—	—	—	—	—	—	—	—	—	—	—	—	—
>25–40	1.3	1.0	1.7	1.0	0.8	1.4	1.0	0.8	1.2	0.9	0.8	1.1	1.1	0.7	1.8	1.1	0.7	1.8
>40–65	1.9	1.5	2.5	1.3	0.9	1.8	1.5	1.2	1.8	1.4	1.1	1.8	1.2	0.8	1.8	1.1	0.7	1.7
>65	2.9	1.5	5.6	2.3	1.1	4.7	2.1	1.2	3.7	2.2	1.2	4.0	1.8	1.1	2.7	1.3	0.8	2.1
*Allocated primary care physicians that are clinically active (PCSA)/100,000 population*																		
<57	1.9	1.4	2.5	1.4	1.0	1.9	2.2	1.7	2.9	1.6	1.3	2.1	1.1	0.8	1.5	1.0	0.8	1.4
57–82	1.6	1.3	2.0	1.4	1.1	1.8	1.5	1.2	1.9	1.2	1.0	1.6	1.1	0.8	1.4	1.0	0.8	1.3
>82	—	—	—	—	—	—	—	—	—	—	—	—	—	—	—	—	—	—
*Medically underserved area*																		
Not a MUA/P	—	—	—	—	—	—	—	—	—	—	—	—	—	—	—	—	—	—
Med. unders. area	1.5	1.2	2.0	1.1	0.8	1.4	1.6	1.4	2.0	0.9	0.7	1.1	0.9	0.7	1.2	0.8	0.6	1.1
Med. unders. pop.	1.4	1.0	2.0	1.1	0.8	1.6	2.2	1.3	3.7	1.3	0.7	2.1	2.0	1.6	2.6	1.2	0.9	1.6
*Segregation*	1.0	0.9	1.0	0.9	0.9	1.0	0.9	0.9	1.0	0.9	0.9	1.0	1.0	1.0	1.0	0.9	0.9	1.0
