# Supplementary figures and images for: The Role of Neighborhood Characteristics in Late Stage Melanoma Diagnosis among Hispanic Men in California, Texas, and Florida, 1996–2012
Source: J Cancer Epidemiol. 2017 Jun 18;2017:8418904. doi: 10.1155/2017/8418904 (PMC5494113; doi:10.1155/2017/8418904)

A

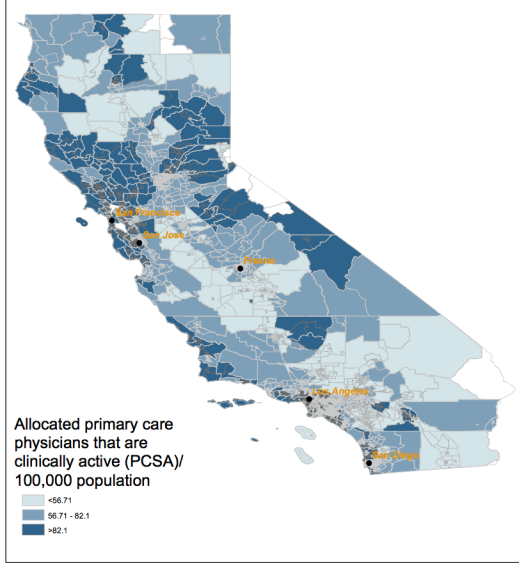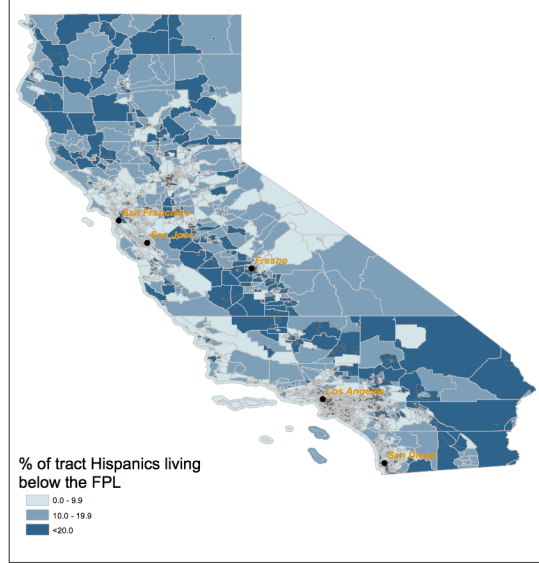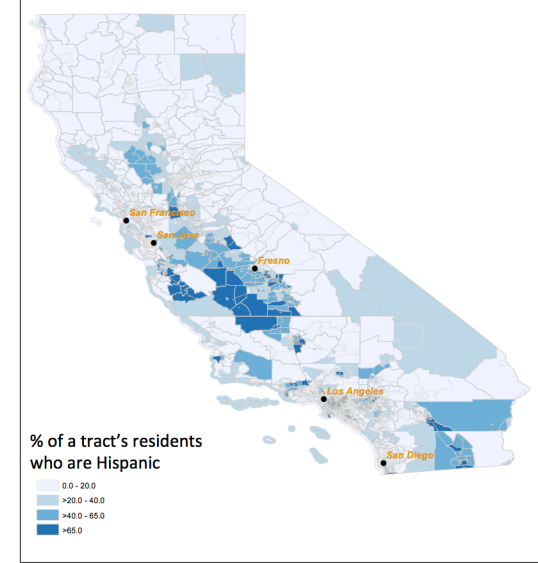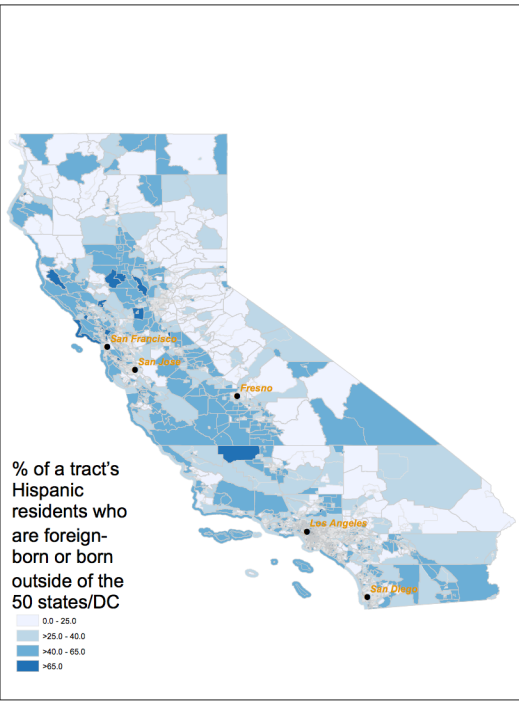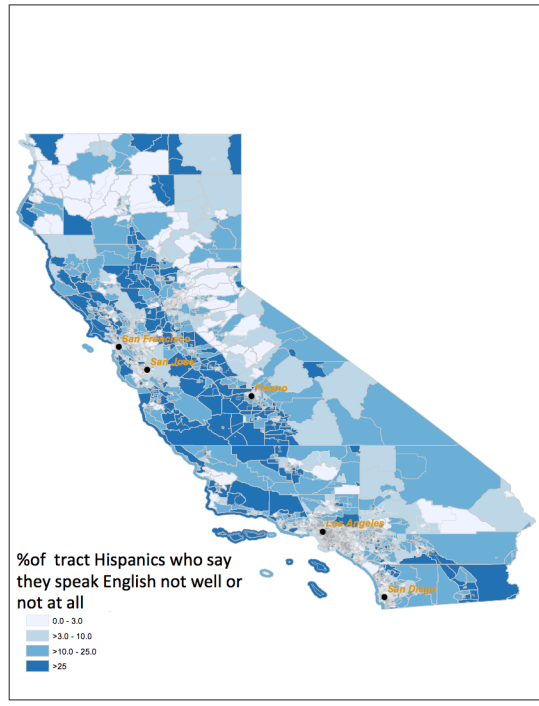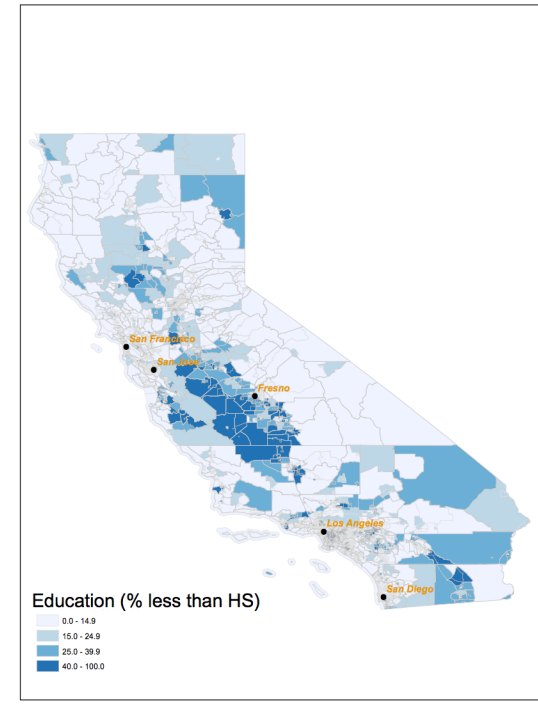

B

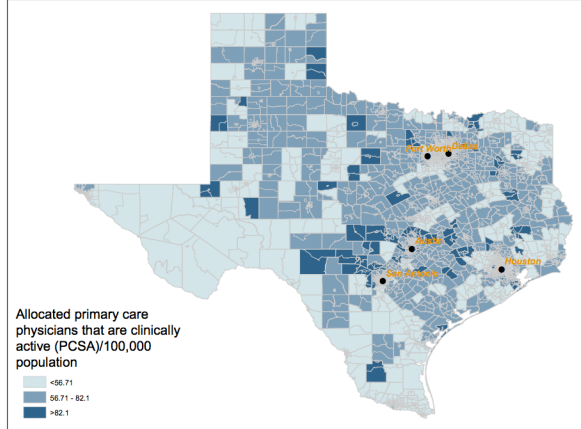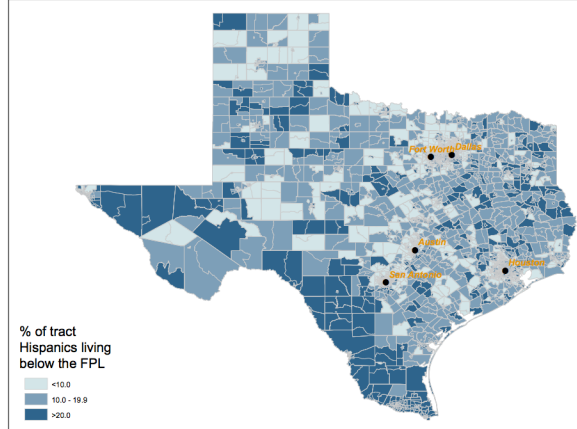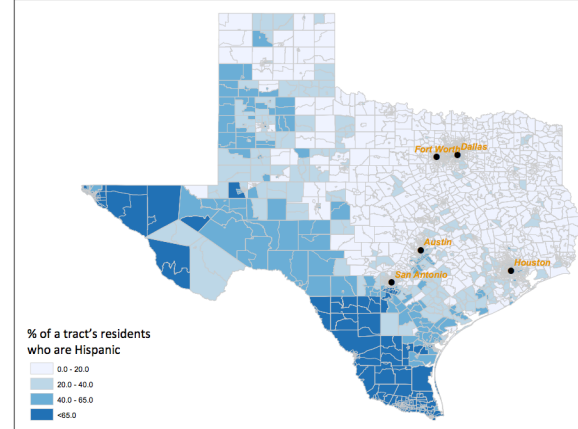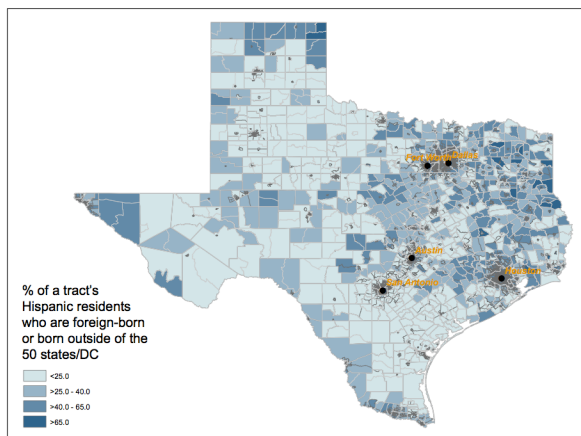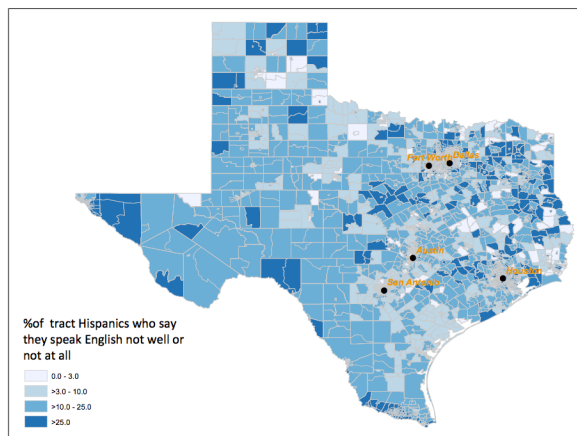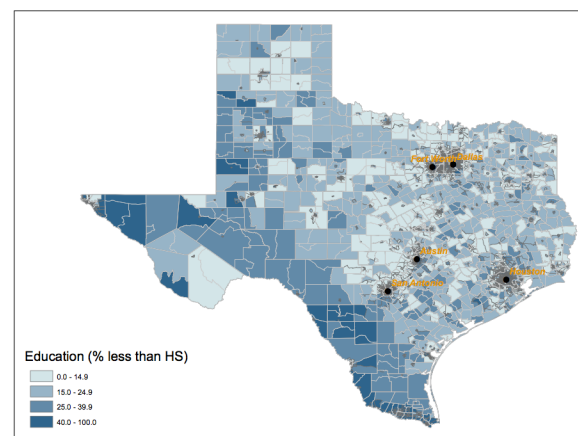

C

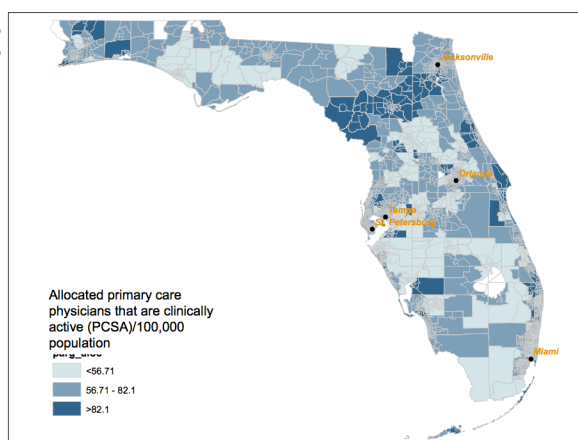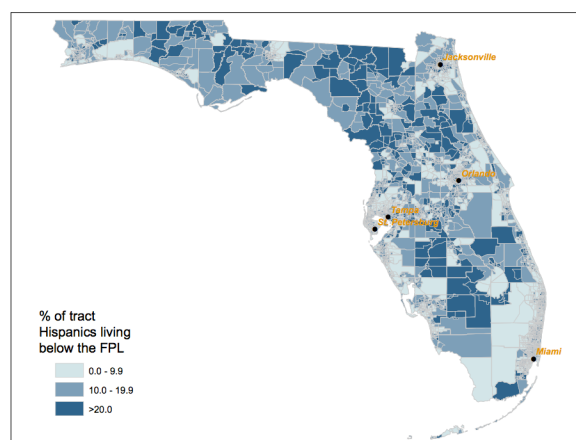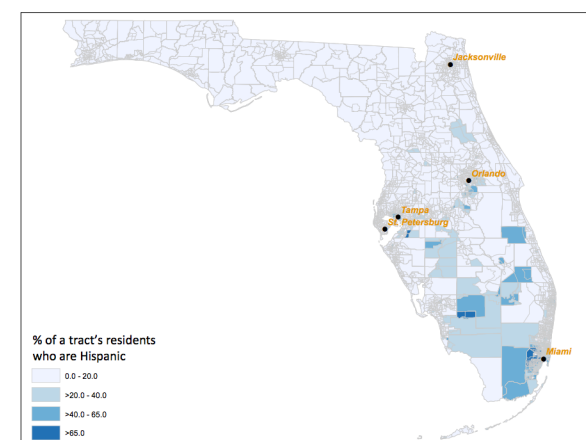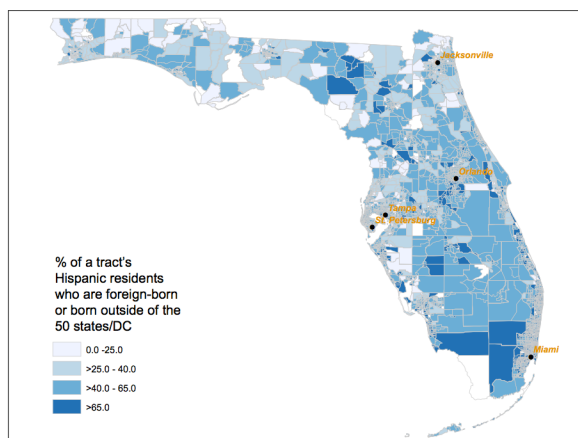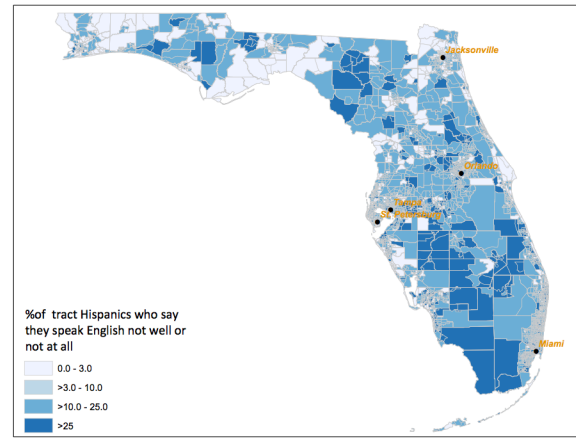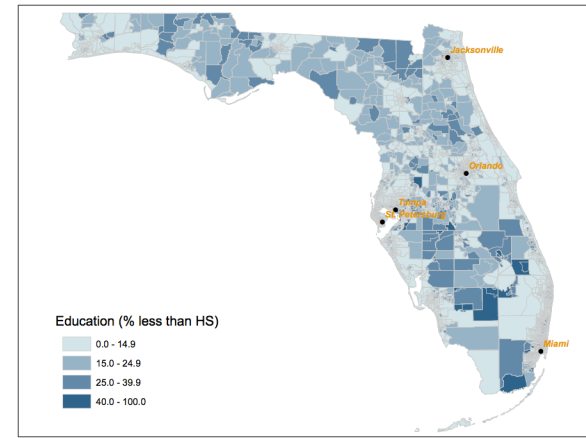

Supplement: Supplementary file 1 — Supplemental Figure 1. Spatial distribution of contextual characteristics in California, Texas, and Florida. [file 8418904.f1.pdf]
